# Supplementary material for: Hydrotalcite–Niclosamide Nanohybrid as Oral Formulation towards SARS-CoV-2 Viral Infections
Source: Pharmaceuticals (Basel). 2021 May 19;14(5):486. doi: 10.3390/ph14050486 (PMC8160721; doi:10.3390/ph14050486)
Supplement: Supplementary file 1 [file pharmaceuticals-14-00486-s001.zip › pharmaceuticals-1188508-supplementary.pdf]

pharmaceuticals

Supplementary Materials

Hydrotalcite-Niclosamide Nanohybrid as Oral Formulation towards SARS-CoV-2 Viral Infections

Goeun Choi [1,2,3], Huiyan Piao [1], N. Sanoj Rejinold [1], Seungjin Yu [1,4], Ki yeok Kim [5], Geun-woo Jin [5,*] and Jin-Ho Choy [1,6,7,*]

1 Intelligent Nanohybrid Materials Laboratory (INML), Institute of Tissue Regeneration Engineering (ITREN), Dankook University, Cheonan 31116, Korea; 12192032@dankook.ac.kr (H.P.), sanojrejinold@dankook.ac.kr (N.S.R.)
2 College of Science and Technology, Dankook University, Cheonan 31116, Korea; goeun.choi@dankook.ac.kr
3 Department of Nanobiomedical Science and BK21 PLUS NBM Global Research Center for Regenerative Medicine, Dankook University, Cheonan 31116, Korea
4 Department of Chemistry, College of Science and Technology, Dankook University, Cheonan 31116, Korea; 32182808@dankook.ac.kr
5 R&D Center, CnPharm Co., LTD., Seoul 03759, Korea; kyeoksky@cnpharm.co.kr
6 Department of Pre-medical Course, College of Medicine, Dankook University, Cheonan 31116, Korea
7 Tokyo Tech World Research Hub Initiative (WRHI), Institute of Innovative Research, Tokyo Institute of Technology, Yokohama 226-8503, Japan
* Correspondence: geunwoo.jin@cnpharm.co.kr (G.J.); jhchoy@dankook.ac.kr (J.-H.C.)

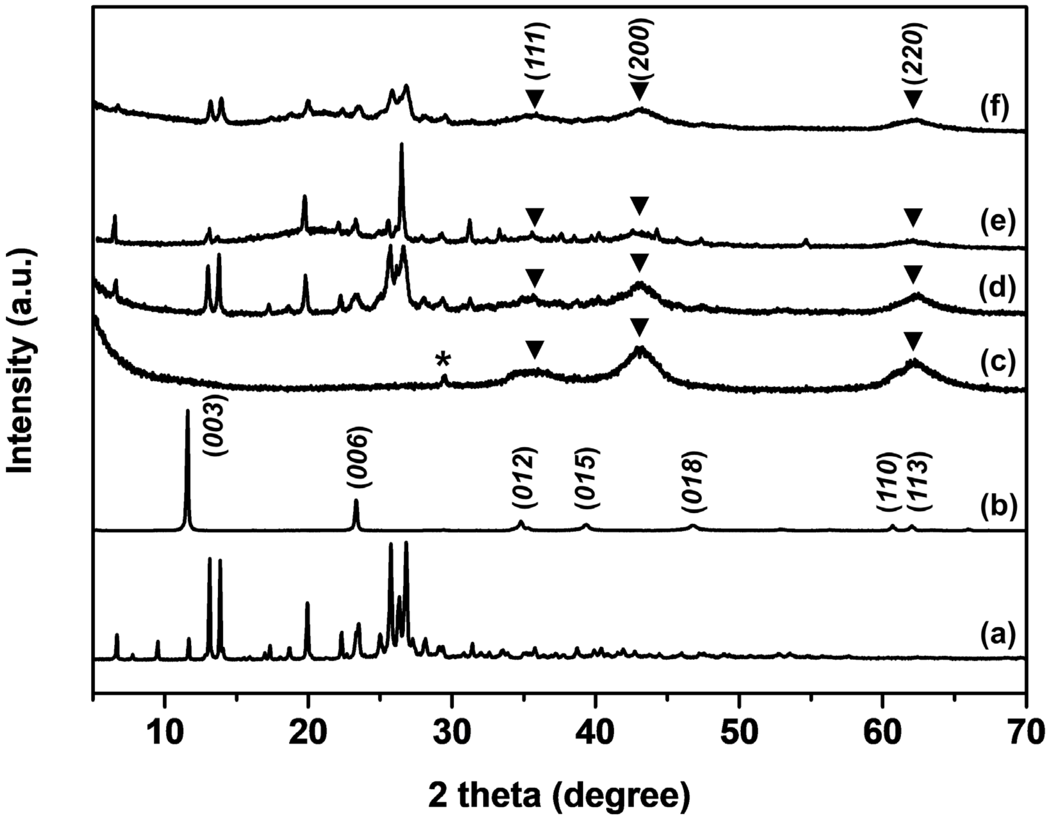

Figure S1. X-ray diffraction patterns of (a) NIC, (b) HT, (c) DHT, (d) NIC-DHT, (e) NIC-DHT/Tween 60, and (f) NIC-DHT/HPMC. (*; impurity, ▼; periclase (MgO)).

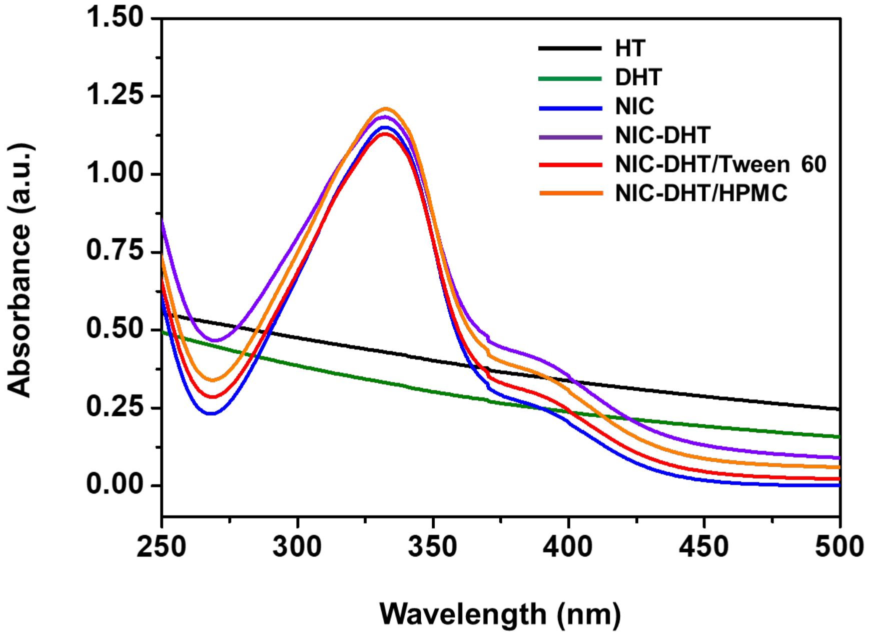

Figure S2. UV–visible absorption spectra of HT, DHT, NIC, NIC-DHT, NIC-DHT/Tween 60, and NIC-DHT/HPMC hybrids in ethanol.

According to the UV–Visible absorption analysis, we observed the characteristic peak of pure NIC in ethanol at 333 nm. This characteristic NIC peak was retained in both NIC-DHT and NIC-DHT/Tween 60 nanohybrid without any specific shift in the absorption band, indicating that Tween 60 was coated on the surface of NIC-DHT via a simple physical adsorption, that it was not chemically modified on the same.

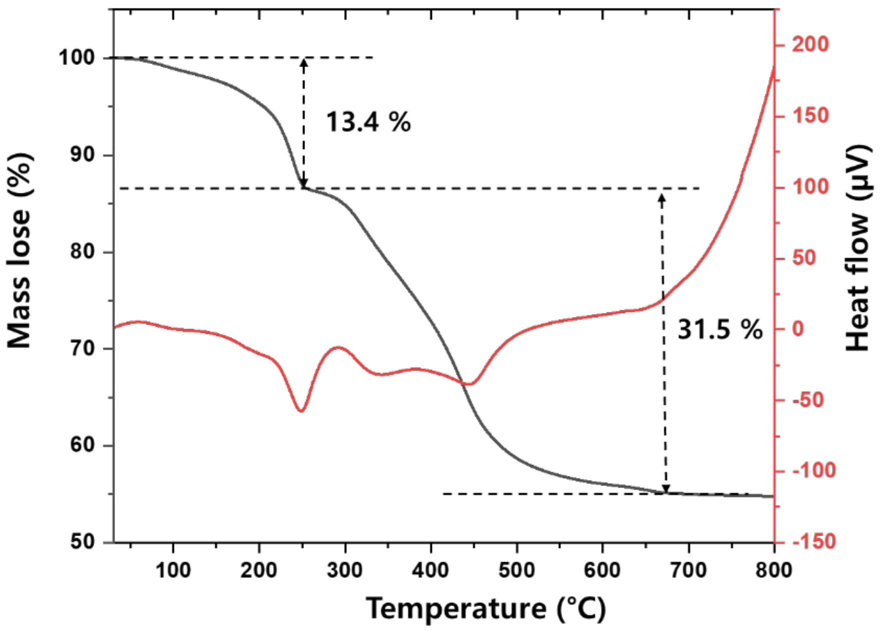

Figure S3. TGA and DTA curves for HT (Hydrotalcite).

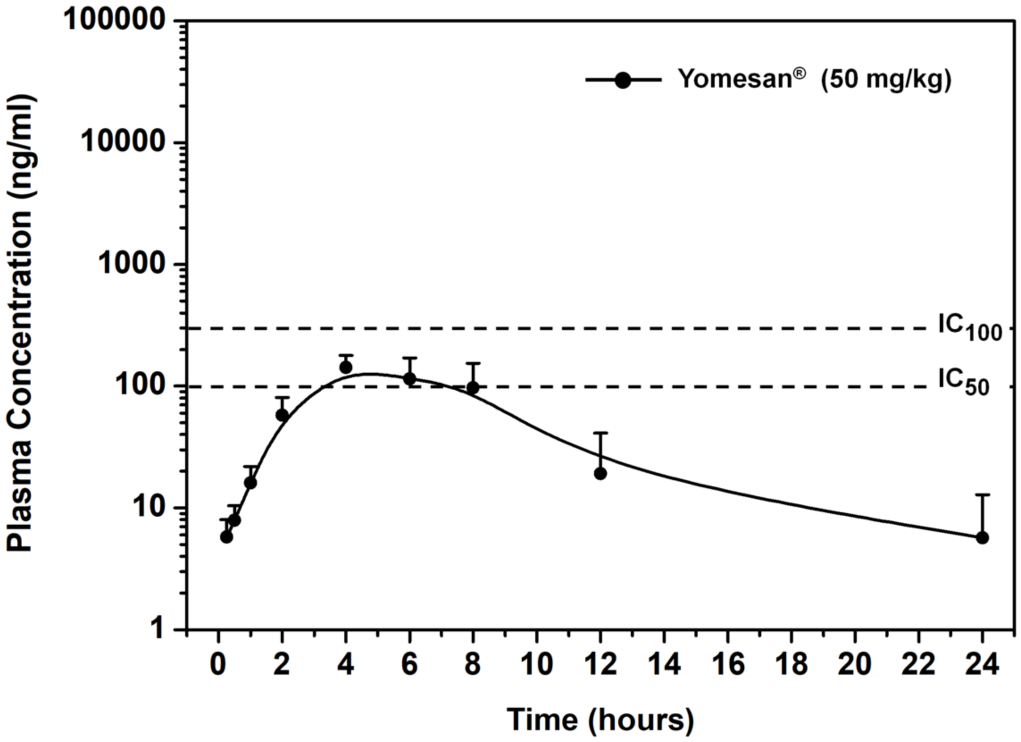

Figure S4. Plots of mean plasma concentration for NIC versus time for Yomesan®. This data shown here is reused under Creative Commons Attribution (CC BY) license (http://creativecommons.org/licenses/by/4.0/).[1]

Reference

1. Yu, S.; Piao, H.; Rejinold, N.S.; Jin, G.; Choi, G.; Choy, J.-H. Niclosamide–Clay Intercalate Coated with Nonionic Polymer for Enhanced Bioavailability toward COVID-19 Treatment. *Polymers* **2021**, *13*, doi:10.3390/polym13071044.

Determination of NIC content: NIC contents were evaluated based on the formula as described below.

$$\text{NIC contents (\%)} = \frac{\text{Weight of NIC (mg)}}{\text{Net weight of formulation (mg)}} \text{ X } 100$$

NIC contents were calculated by dividing the amount (weight) of NIC acquired by HPLC with the net weight of NIC-DHT, NIC-DHT/Tween60 or NIC-DHT/HPMC. The amount of NIC was evaluated by the average value of three HPLC measurements. The NIC calibration curve was validated based on the linearity (R^2) and relative standard deviation (RSD) as shown below.

- $R^2 = 0.999$
- RSD (%) of the slope = 0.46 %
- LOQ = 0.828 µg/mL

Trial 1

	Concentration (ug/mL)	Peak area
1	70.00	2882
2	60.00	2471
3	50.00	2055
4	40.00	1660
5	30.00	1262
6	20.00	852
7	10.00	445

Slope	**38.65**
Intercept	**40.44**
Correlation coefficient (r^2)	**0.99996**

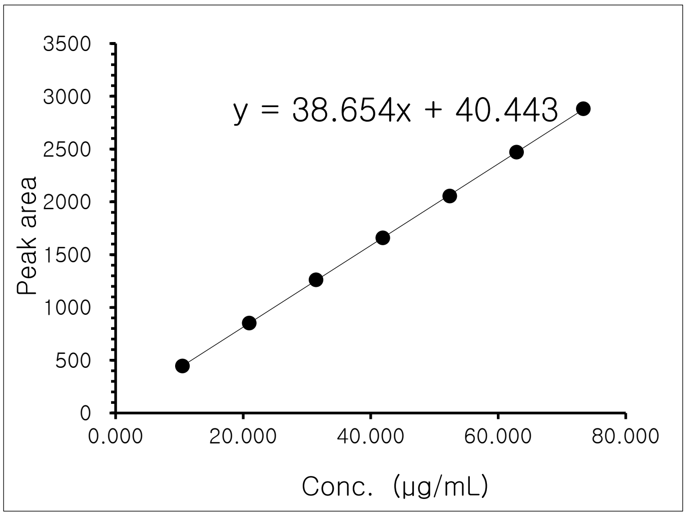

Trial 2

	Concentration (ug/mL)	Peak area
1	70.00	2703
2	60.00	2399
3	50.00	1996
4	40.00	1589
5	30.00	1214
6	20.00	816
7	10.00	417

Slope	**38.59**
Intercept	**46.79**
Correlation coefficient (r^2)	**0.99901**

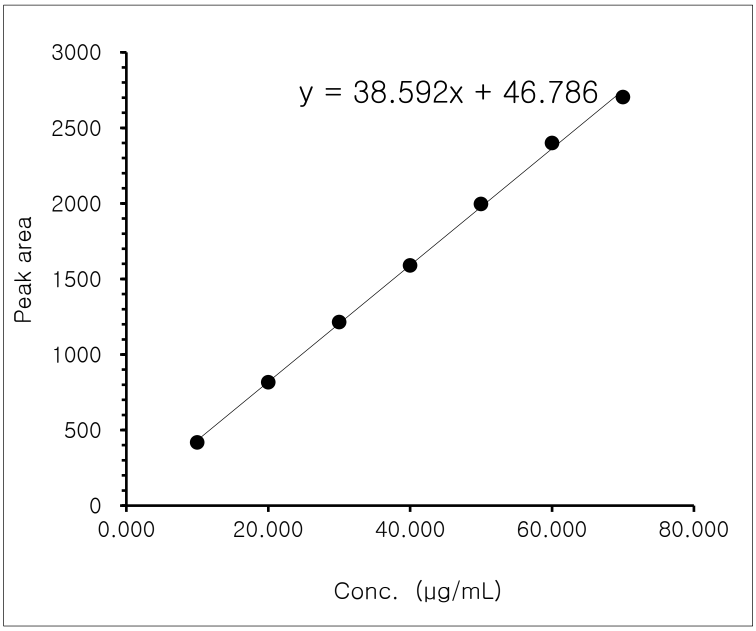

Trial 3

	Concentration (ug/mL)	Peak area
1	70.00	2993
2	60.00	2572
3	50.00	2169
4	40.00	1732
5	30.00	1325
6	20.00	883
7	10.00	459

Slope	**38.32**
Intercept	**44.26**
Correlation coefficient (r^2)	**0.99989**

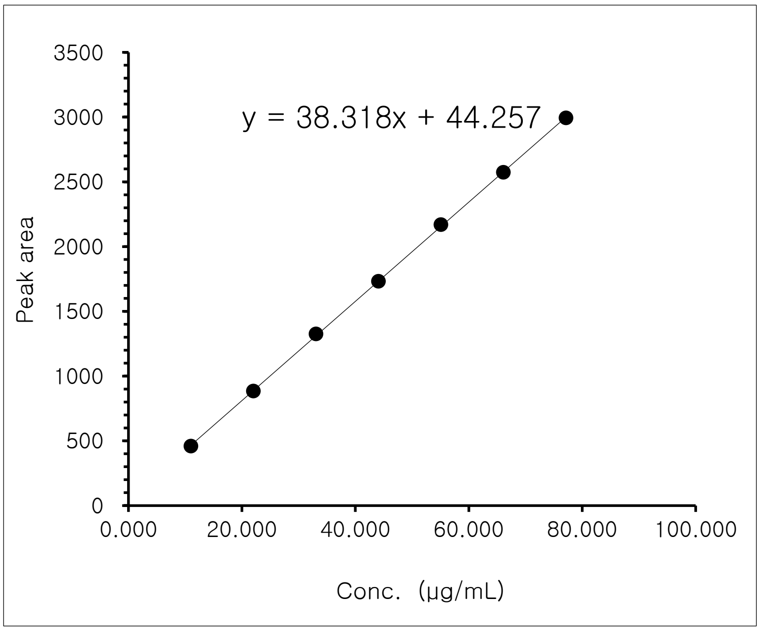

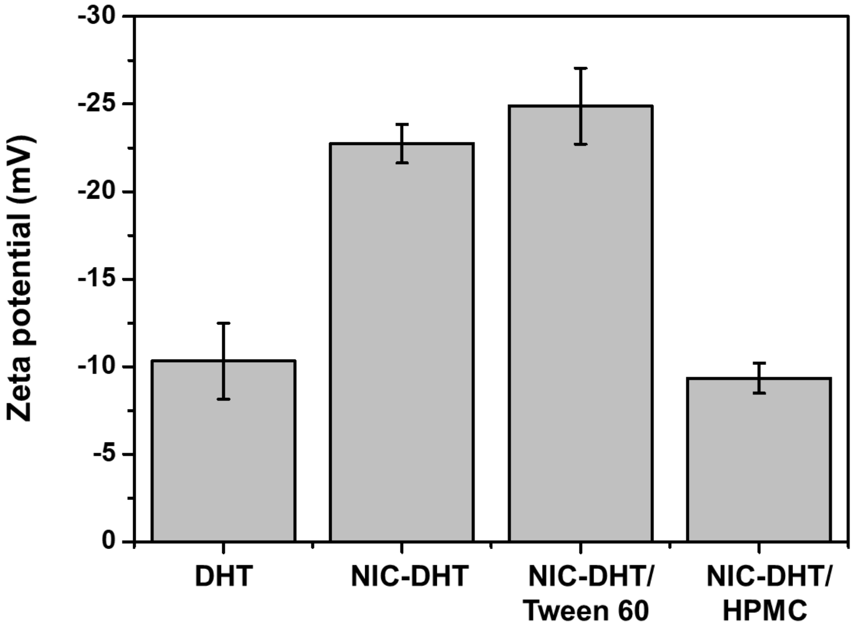

Figure S5. The surface charges (ξ-potential) of DHT, NIC-DHT, NIC-DHT/Tween 60, and NIC-DHT/HPMC (n=3).

The zeta potential of DHT, NIC-DHT, NIC-DHT/Tween 60, and NIC-DHT/HPMC was determined to be -10.33 ± 2.17 mV, -22.74 ± 1.10 mV, -24.89 ± 2.16 mV, and -9.35 ± 0.86 mV, respectively. Such negative potantials observed for the polymer coated are quite reasonable, since the negatively charged NIC-DHT was coated with non-ionic polymer.
